# *Thyroid Research* celebrates its 15th year of publication achieving its first Journal impact factor

**DOI:** 10.1186/s13044-023-00179-z

**Published:** 2023-09-01

**Authors:** Simone de Leo, Bijay Vaidya

**Affiliations:** 1 Department of Endocrine and Metabolic Diseases, Endocrine Oncology Unit, IRCCS Istituto Auxologico Italiano, Milan, Italy; 2 Department of Endocrinology, Royal Devon & Exeter Hospital, University of Exeter Medical School, Exeter, UK

In the last 15 years, thyroid knowledge has evolved significantly. Management of thyroid cancers has dramatically changed with the increasing use of active surveillance in small thyroid tumors, the more tailored use of radioactive iodine treatment, and the development of tyrosine kinase inhibitors in the treatment of advanced thyroid cancers. The better understanding of the mechanisms involved in thyroid dysfunction and the identification of genetic basis for different thyroid disorders has not only improved the preoperative management of thyroid nodules but also that of advanced thyroid cancers, with the use of targeted compounds based on molecular genetic findings. Monoclonal antibody drugs have been developed for thyroid eye disease, marking a paradigm shift in the treatment of this disease, and studies are in progress for new treatments for Graves’ disease. The impact of thyroid dysfunction on fertility and pregnancy outcomes is now well reported; however, management of different thyroid disorders in women planning pregnancy or pregnant women remains a largely debated topic.

*Thyroid Research* has covered all these topics in its 15 years of life, playing an important role in advancing the frontiers of thyroid knowledge and promoting its dissemination. In these 15 years, *Thyroid Research* has published more than 350 papers, with some of them being cited more than 150 times [1, 2] and downloaded more than 85,000 times [1, 3, 4]. In the last 5 years, *Thyroid Research* has received submissions from 59 countries across 6 continents, making it a truly international journal. This year, we introduced a thematic series on the thyroid in pregnancy and infertility (https://www.biomedcentral.com/collections/TTPI). This series will provide our readers and the scientific community an in-depth analysis on this theme, with expert authors in the field invited to contribute to the series. The importance of dissemination of thyroid knowledge is guaranteed by the Open Access format of the Journal. This permits an immediate and free access to all researchers and people around the world interested in the thyroid field (physicians, biologists, healthcare providers etc.), breaking down economic or bureaucratic barriers and ensuring that groundbreaking discoveries reach even the most remote corners of the globe.

In the 15 years, the Editorial Board (including Associate Editors) of *Thyroid Research* has been renewed with the involvement of early career researchers, with outstanding curricula, that accompany the more senior researchers. This process is still ongoing; it will help us to offer an improved review process, with a better experience for authors submitting to the Journal.

We would like to thank everyone who helped to achieve the goals of *Thyroid Research*: Associate Editors, Editorial Board members, Reviewers, all the researchers who chose our Journal for their papers, BioMed Central, Springer Nature, and all our readers. Even though we celebrate *Thyroid Research’s* first 15 years of life and its first impact factor today, we recognize that our journey has only just begun. Our attention is already directed on our future goals: in particular, improving impact factor and other journal metrics, which will make *Thyroid Research* one of the favorite journals for thyroidologists and endocrinologists around the World.
